# Flexural Behaviour of Cementitious Mortars with the Addition of Basalt Fibres

**DOI:** 10.3390/ma14061334

**Published:** 2021-03-10

**Authors:** Małgorzata Wydra, Piotr Dolny, Grzegorz Sadowski, Jadwiga Fangrat

**Affiliations:** 1 Faculty of Civil Engineering, Mechanics and Petrochemistry, Warsaw University of Technology, 09-400 Płock, Poland; piotr.dolny@pw.edu.pl (P.D.); grzegorz.sadowski@pw.edu.pl (G.S.); 2 Building Research Institute, 00-611 Warsaw, Poland; j.fangrat@itb.pl

**Keywords:** basalt fibres, cementitious mortar, flexural strength, bridging effect

## Abstract

The results of flexural tests of basalt fibre-reinforced cementitious mortars in terms of flexural strength and the occurrence of the bridging effect are summarised. Mixture proportions and curing conditions were altered for various series. The main parameters concerning mixture proportions were water to cement ratio (*w/c*), micro-silica and plasticiser addition and fibre dosage (1%, 3% and 6.2% by binder’s mass). Various curing conditions were defined by different temperatures, humidity and time. The influence of the amount of water inside the pores of the hardened cementitious matrix on the flexural strength values, as far as the impact of the alkaline environment on basalt fibres’ performance is concerned, was underlined. The designation of flexural strength and the analysis of post-critical deformations were also performed on the reference series without fibres and with the addition of more common polypropylene fibres. The bridging effect was observed only for the basalt fibre-reinforced mortar specimens with a relatively low amount of cement and high *w/c* ratio, especially after a short time of hardening. For the lowest value of *w/c* ratio (equalling 0.5), the bridging effect did not occur, but flexural strength was higher than in the case of non-reinforced specimens. Comparing mortars with the addition of basalt and polypropylene fibres, the former demonstrated higher values of flexural strength (assuming the same percentage dosage by the mass of the binder). Nevertheless, the bridging effect in that case was obtained only for polypropylene fibres.

## 1. Introduction

The performance properties of traditional construction mortars (including cementitious mortars) are based primarily on the specific proportions among the binder, the fine aggregate and water. Their high compressive strength makes them suitable as binding material in connections of two structural elements, such as brick to brick or any end connection of beam and column. Their limitations in such applications, however, result mostly from their brittle behaviour and the ease of cracking because of shrinkage, concentration of stresses, low tensile strength and weakness in impact resistance [1]. Despite these disadvantages, user demand is growing, which enforces the continuous development of mortar technology and production [2].

Fibre addition is thought to overcome some of these limitations due to their beneficial influence on the mechanical properties of hardened mortar, such as impact resistance, and splitting tensile and flexural strengths [3,4,5,6,7]. Additionally, the improvement of tensile strength, flexural toughness and energy absorption capacity due to fibre addition, as far as the mechanics of cracking propagation are concerned, have been confirmed in the case of the most popular cementitious material—concrete—in numerous experimental [8,9,10,11,12,13,14,15,16,17,18,19,20,21,22] and numerical [23,24,25,26,27] investigations. The latest research programmes in that area are focused mostly on subjects concerning newly developed materials [8,10,20], the ecological impact of building materials [19,22] or less typical performance tests [18,21]. Moreover, the influence of various aggressive environments on fibre-reinforced concrete has been described in articles [28,29]. Fibre-reinforced material investigations are also important in terms of the growing interest in fibre-reinforced polymer composites [30,31].

The reduction in crack width and number of cracks in hydraulic binders’ matrices is related to the so-called bridging effect, which involves transferring stresses from a cracked mortar layer on intact composite fibres [7,32,33,34,35]. Such reduction in crack width and the number of cracks due to fibre usage might be useful in terms of the durability performance of the material, as deleterious substances (such as water, chlorides and carbon dioxide) have limited possibility of ingression into the concrete construction element [15]. The phenomenon has already been confirmed in the case of concrete with newly developed basalt fibres [8]. The mechanism of the bridging effect can be described as follows: When the maximum value of stresses is gained in a flexural test, the first damages appear and the cracking process begins. Fibres inside these cracks, however, form a kind of bridge between the separated edges of the cracked matrix and can still transfer part of the stresses. As a result, crack width enlarges gradually, the development of macro cracks is delayed and tensile strength is improved [32].

The improved bond behaviour of fibre-reinforced cementitious composites makes them suitable not only for newly constructed elements but also for the repairing of existing constructions [4,36,37]. As the behaviour of cementitious mixtures during their implementation is also an important matter and may influence potential applicational areas, the parameters concerning the testing of fresh mixtures with fibre additions have already been the subject of numerous investigations, including [4,32,38,39,40].

Fibres that are most commonly used to reinforce mortars are made of steel, carbon, glass, polypropylene and basalt [41,42,43,44], although the use of other natural fibres, such as sisal, has also been reported [45]. As the basalt fibres used in this study are a relatively new material [46,47,48,49,50], the properties of composite materials (including cementitious mortars) with their addition have not been fully discovered yet, and intensive research is still recommended [42]. A summary of the studies carried out thus far on various types of mortars with the addition of basalt fibres was presented in [51], where the authors suggested the following. “The various proportions of raw materials, necessary test methods and compliance with the desired standards for various properties like fresh, mechanical, durability and dynamic properties of fibre-reinforced mortar helps to conclude its practical applicability in civil engineering applications.”

The ecological impact of cementitious materials, due to their high natural resource consumption, is the subject of intensive discussion [52,53]. It should be noted, however, that basalt fibres are known to be an ecologically friendly material [51]. The production of basalt fibres requires less cost and energy with respect to commercially available synthetic fibres, while their performance, based on recent investigations, is found to be comparable. Additionally, they indicate no chemical reaction when in contact with some chemicals. Multiple potential applications of basalt fibre-reinforced cementitious materials are currently a subject of discussion in terms of sustainable development [7,50,54].

However, low resistance to an alkali environment inside a cementitious matrix should not be neglected when analysing potential applicational areas of analysed composite material [3,40,47]. The resistance of basalt fibres to alkali solution was investigated in the article [55]. Although the loss of mass of basalt fibres appeared to be sensitive to the concentration of alkali and for a lower alkalinity mass of fibres remained almost unaffected, the tensile strength of fibres was reduced significantly, regardless of the concentration of the alkali.

**Figure 1:**
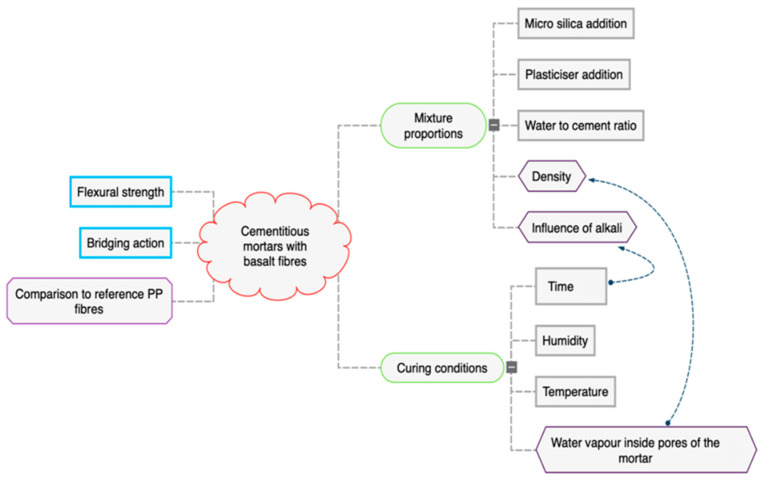
Mind map presenting the concept of this study.

Within the current study, the flexural performance of cementitious matrices with the addition of basalt fibres in terms of flexural strength and the bridging effect was analysed. The planned series of specimens differed in terms of mixture proportions and curing conditions. Mortars without the addition of fibres and with the addition of more typical polypropylene (PP) fibres were used as a reference. A mind map presenting the concept of this study is shown in Figure 1.

The main research problems to consider were as follows:

- Will the bridging effect occur in cementitious mortars with the addition of the recently developed basalt fibres, as it has been discovered for other fibre-reinforced materials (e.g., concrete)?
- Will the addition of basalt fibres to cementitious mortar change the value of flexural strength?
- How do the curing conditions’ parameters (humidity, time and temperature) influence the flexural performance of cementitious mortars with the addition of basalt fibres?
- Is there any relation between the mixture proportions parameters (such as water to cement ratio, fibre dosage, presence of micro-silica and plasticiser) and the flexural performance of cementitious mortars with the addition of basalt fibres?

The investigation presented herein intended to provide answers to these research questions for the proposed and analysed mixture proportions of cementitious mortars with basalt fibres. In the authors’ opinion, the presented results of this experimental program can be generalized and, therefore, enable the proper definition of end users’ benefits.

The motivation behind the use of the above-mentioned additions in these experiments was the fact that the addition of micro-silica with an adequate amount of plasticiser has already been proven to provide higher values of stresses in pull-out tests due to the stronger bond between various types of fibres and cementitious matrices with micro-silica addition [56,57]. Additionally, the applicational potential of nanosilica in cement composites has been underlined in article [58].

Regarding curing conditions, the water accessibility during the hardening processes of the cement matrix and intensity of water vapour pressure inside the pores during the test might influence the values of experimentally designated flexural strength.

Additionally, the density of the specimens (including the amount of water inside pores) for each series was established, as various mixture proportions and curing conditions might result in different values of the mortars’ specific density and mass of free water inside the pores.

On the basis of the analysis of experimental results, the authors concluded that the resistance of basalt fibres to the alkali environment inside the cementitious matrix over time might influence the flexural behaviour of the basalt fibre-reinforced cementitious matrix, which should be investigated more carefully in future research programmes. The above statement is in line with the conclusions drawn by other researchers [3,40,47].

Although the bridging effect is a widely known phenomenon, especially in the case of fibre-reinforced concrete [16], the concern of this research programme is its occurrence in a recently developed material, basalt fibre-reinforced cementitious mortar. Apart from positive influence of basalt fibres on the flexural performance of cementitious mortars observed for several series, challenges that need further consideration are highlighted.

## 2. Materials

Portland cement CEM I 32.5R was used to prepare cement mortar mixes. Sand was used as fine aggregate fraction at 0–2 mm in accordance with the EN 196–1 standard [59]. Tap water was added as mixing water. For specific series, micro-silica and plasticiser were used. Basalt chopped fibres (BCFs) were used for the “BCF” series, while polypropylene fibres were used for the “PP” series. Series designated with “W” were prepared without the addition of fibres.

The BCF were 12 mm long and about 13 µm in diameter. The density of the basalt fibres was 2.6–2.8 g/cm^3^, while the modulus of elasticity was in the range of 91 to 110 GPa. As declared by the manufacturer, the soaking rate of the BCFs used was less than 0.1%, and their melting point equalled to 1450 °C. Their chemical composition is shown in Table 1.

Polypropylene (C_3_H_6_)_n_ fibres (designated later in the article as PP) used in this experimental programme were 12 mm long and 20 µm in diameter. The density of the PP fibres was approximately 0.91 g/cm^3^, the modulus of elasticity was 3.8 GPa and the tensile strength was about 360 MPa. Polypropylene fibres are characterised by elongation at a tension of about 20%, a melting point of about 145 °C, high chemical resistance and non-absorbability.

**Figure 2:**
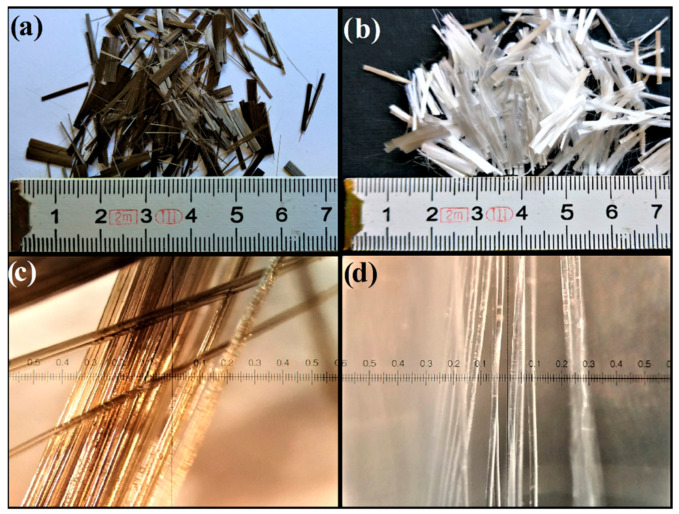
Basalt and polypropylene fibres: (**a**) basalt fibres on a real scale (cm), (**b**) polypropylene fibres on a real scale (cm), (**c**) basalt fibres magnified (0.1 × mm), (**d**) polypropylene fibres magnified (0.1 × mm).

Figure 2a,b show basalt and polypropylene fibres on a real scale, while Figure 2c,d show a magnification of the described fibres.

The bulk density of the used micro-silica was 650 kg/m^3^, and its chemical composition, as declared by the manufacturer, is shown in Table 2.

Three samples with dimensions of 40 × 40 × 160 mm were submitted for testing for each series. The series differed in terms of mixture proportions (such as water to cement ratio, the addition of plasticiser and micro-silica and the type and dosage of used fibres), the time elapsed between the date of sampling until the tests and the curing conditions. The recipes are summarised in Table 3, while the curing conditions are described later on in this section. In this research programme, the basalt fibre doses for various series were 1%, 3% and 6.2% by the mass of the binder, while the authors of article [51] suggested, on the basis of recent studies, that the proper ratio is 1–3%.

The samples were prepared and tested in accordance with EN 196-1:2016 [59]. The fibres were dry mixed with sand and homogenised. The dosing of individual mortar components and the mixing process were carried out using a planetary mixer with a programmed schedule according to the standard [59]. After mixing, the mortar was laid in two layers in a steel mould with a capacity for three specimens. After each layer was laid, compaction was performed in a cycle of 60 strokes within 60 s. It was noticed that fresh mortar with the addition of basalt fibres was visibly denser than the reference mixtures without fibres. The mortar specimens were levelled, covered with glass plates placed at specific distances and stored in the moulds for 24 or 48 h (depending on the series type) in a room with air temperature of 20 °C. Further curing conditions of the samples and the storage time of individual series are shown in Table 4.

## 3. Methods

### 3.1. Flexural Strength Tests

**Figure 3:**
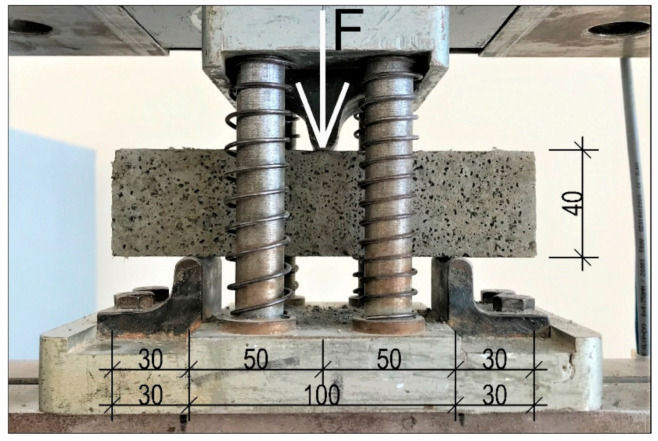
Test stand, dimensions in mm.

No less than three specimens of each mortar type were subjected to three-point bending tests in order to obtain their flexural strength. The tests were carried out in a 100 kN capacity hydraulic press. The test stand and static scheme are shown in Figure 3.

Each dimension of the specimen was measured with the use of a digital calliper (with accuracy of 0.01 cm) before placing it on the test stand. Specimens were submitted to static load with the speed ratio maintained at 1.5 mm/min. The maximum values of the load were determined for each specimen, and then flexural strength *f_cf_* was calculated. Analysis of the bridging effect during the post-critical stadium (after gaining the maximum value of load) is especially important in the case of fibre-reinforced mortars. In this article, authors present their organoleptic observations during the tests of post-critical behaviour for the specimens of series W3_28D, BCF3_28D, PP3_28D, W3_28D_70, BCF3_28D_70 and PP3_28D_70. For the specimens of series W1_21D, BCF1_21D, W2_1D, BCF2_1D, W2_21D and BCF2_21D, additional analysis was performed, as authors decided to registrate the dependence between the values of external force and displacement of the machine’s loading plates in order to prepare and analyse load-displacement and stress–strain curves. Fracture energy was calculated for these series as well.

Displacement was measured as the mutual relocation of hydraulic press holders during the test. Such displacement corresponds to the deflection of the specimen during the test. Strains were then calculated on the basis of the following system of equations (assuming elastic response of homogeneous rectangular cross section under flexure):

(1) $$\left\{\begin{array}{c} \delta =\alpha \frac{M}{B}l^{2}\\ \alpha =\left(\frac{1}{8}-\frac{\xi^{2}}{6}\right)\\ \varepsilon =\frac{M}{B}\frac{h}{2} \end{array}\right.$$

where *δ*—deflection of the element; *M*—current value of internal bending moment of the section’s neutral axis in the central part of the specimen; *B*—stiffness of the cross section; *α*—parameter taking into account static scheme of the specimen; *l*—length between supports during the test; *ξ*—relative distance between applied force and support (for force applied in the middle of the specimen’s length *ξ* = 1/2); *h*—height of the specimen.

Result of above presented system of Equations was as follows:

(2) $$\varepsilon =\frac{6\delta h}{l^{2}}$$

Stresses were calculated analogically to flexural strength, as normal stresses in bent cross-section of beam in four-point bending tests. Finally, fracture energy was determined as per one of two methods presented in [60] using Equation:

(3) $$G_{F}=\frac{W_{0}+mg\delta }{b\cdot h}$$

where *W*_0_—the area under the load–deflection curve; *m*—mass of the specimen; *g*—gravity acceleration; *δ*—deflection under end of the test; *b*—breadth of the specimen; *h*—height of the specimen.

As tests were interrupted for various levels of force, in order to unify analysis for various specimens, a level of 80% of maximum force was assumed as the end of test for each specimen.

### 3.2. Density Determination

All specimens were weighted and measured prior to the flexural tests in order to determine their density as per Equation (4):

(4) $$\rho =\frac{m}{b\cdot h\cdot l}$$

where *m*—mass of specimens after curing as per Table 4, right before the flexural tests; *b*—breadth of the specimen; *h*—height of the specimen; *l*—length of the specimen.

The specimens were weighted with an accuracy of 0.01 g right before the flexural strength tests. The influence of the specimens’ moisture was not taken into account in calculations, so the measured mass includes free water inside the pores overstating the value of pure cement mortar density. Density should then be analysed in terms of whether it is higher or lower for various types of mortars cured in the same conditions (with a similar amount of water vapour inside the pores). The specimens assigned as W3_28D_70, BCF_28D_70 and PP_28D_70, however, were dried out until they reached a stable mass before testing.

## 4. Results

The selected series of cement mortars are analysed below in order to highlight specific aspects noted during the tests.

### 4.1. The Influence of Mixture Proportions

**Figure 4:**
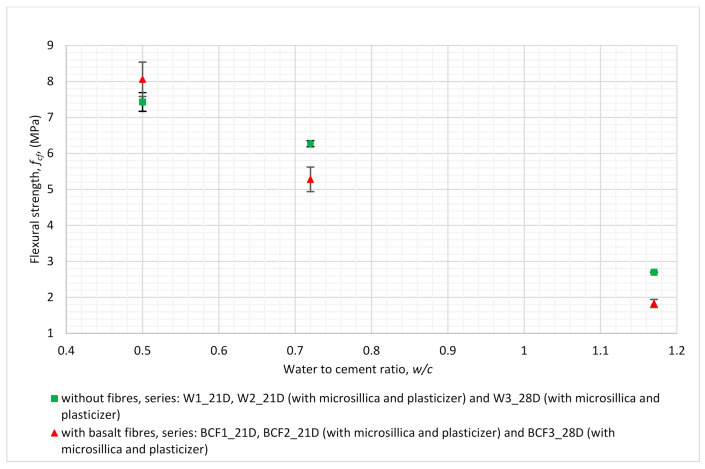
Relation between *w/c* ratio and flexural strength for series revealing the influence of mixture proportions (for detailed descriptions of mixture proportions, see Table 3).

**Figure 5:**
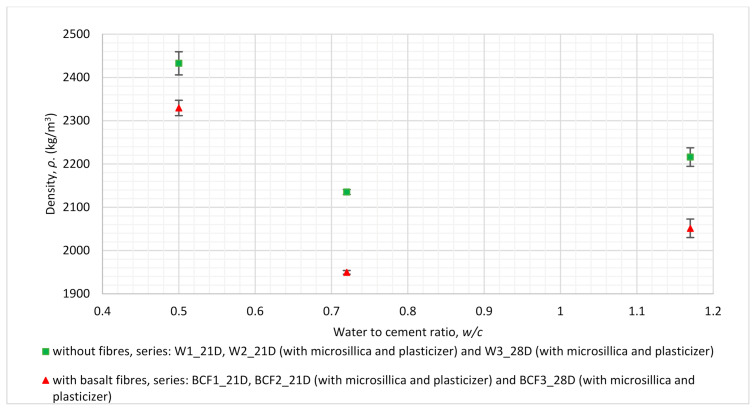
Relation between *w/c* ratio and density for series revealing the influence of mixture proportions (for detailed descriptions of mixture proportions, see Table 3).

The relation between the flexural strength of the mortar and *w/c* ratio for the series analysed in this section is shown in Figure 4, while the dependence between density and *w/c* ratio is presented in Figure 5.

The addition of basalt fibres was efficient in terms of the flexural strengthening of cementitious mortar only in the case of the lowest water to cement ratio, i.e., equal to 0.5 (Figure 4), even though the amount of added fibres was also lowest in that case (5.0 kg/m^3^). It is worth mentioning that micro-silica and plasticiser were used in this mixture proportion (BCF3_28D). Moreover, the addition of basalt chopped fibres had little influence on the descending trend of flexural strength observed with the increase in *w/c* ratio (see Figure 4).

The density decrease observed in Figure 5 for the specimens with basalt fibre addition in reference to non-reinforced specimens can be related to their more porous structure, which is in line with the previously published works of other researchers [6]. The organoleptic observation of the broke hardened specimens revealed a more porous structure in mortars with fibre addition than in those without (cracked interfaces of the specimens after flexural tests are commented in the discussion part). This might correspond to problems with the workability of fresh mortars noted during mortar preparation.

Plasticiser and micro-silica were added for several series in order to obtain better structural integrity (lower porosity, more dense structure, better workability and homogeneity of fresh mixture) for the specimens. This aim was achieved most effectively in the case of specimens designated as BCF3_28D (*w/c* = 0.5), which achieved the lowest difference of density between fibre-reinforced specimens and the reference, non-reinforced specimens (see Figure 5). Higher structural integrity might increase the flexural performance of those specimens (see Figure 4); flexural strength is in that case higher than that of non-reinforced specimens.

### 4.2. The Influence of Specimen Conditioning

**Figure 6:**
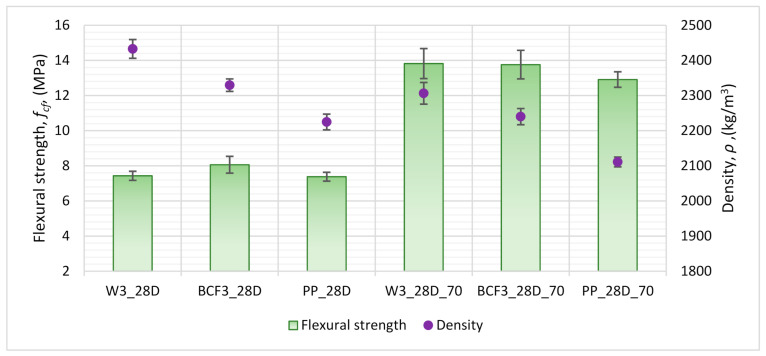
Influence of drying until stable mass on flexural strength and density of cement mortars with various types of fibres (for conditioning descriptions, see Table 4).

The results of flexural strength density (Figure 6) showed how the drying out of specimens until they reach a stable mass increases the values of flexural strength (obtained values up to 86% higher), and how the addition of various types of fibres influence the flexural performance of cementitious mortars in terms of flexural strength value. Apart from basalt fibres, the influence of polypropylene fibres on the properties of cementitious mortars was also analysed.

Mortars with the addition of basalt fibres had a higher value of flexural strength than reference mortars with PP fibres; however, flexural strength only increased for series BCF3_28D in comparison to non-reinforced specimens. The same effect could not be seen in the case of matching dried series BCF3_28D_70. Nevertheless, the loss of flexural strength for this series was on the level of only 0.4% (from 13.82 MPa–W3_28D_70 to 13.76 MPa–BCF3_28D).

Concerning the addition of PP fibres, in both cases (PP3_28D and PP3_28D_70), the obtained values of flexural strength were lower compared to the matching series without fibres (W3_28D and W3_28D_70) and the series with basalt fibres (BCF3_28D and BCF3_28D_70). It was noted, however, that for the PP series, the decrease in strength value after achieving the maximum value during the experiment was slower than in the case of specimens without fibres or with basalt fibres. The deformations kept growing with continuous decrease in strength value after achieving the maximum value of load. A similar observation was reported by other authors (bridging effect [32,33]). For series BCF3_28D and BCF3_28D_70, the bridging effect did not occur. As a result, other cement mortar mixture proportions were proposed in order to perform additional analyses of this phenomenon, as described in Section 4.3.

### 4.3. Post-Critical States Analysis

**Figure 7:**
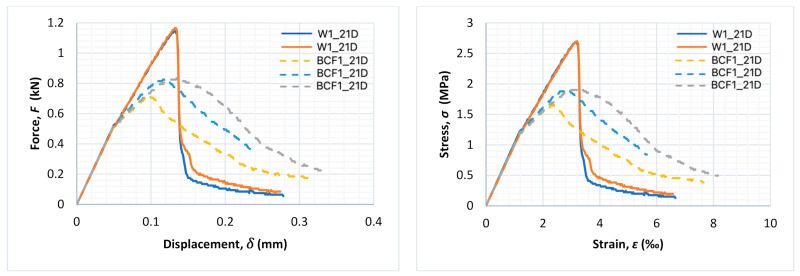
Force–displacement and stress–strain curves for the entire test duration for each specimen of series W1_21D (continuous line) and BCF1_21D (dashed line).

**Figure 8:**
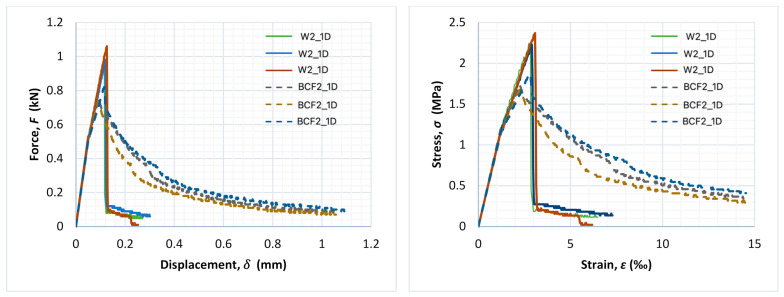
Force–displacement and stress–strain curves for the entire test duration for each specimen of series W2_1D (continuous line) and BCF2_1D (dashed line).

**Figure 9:**
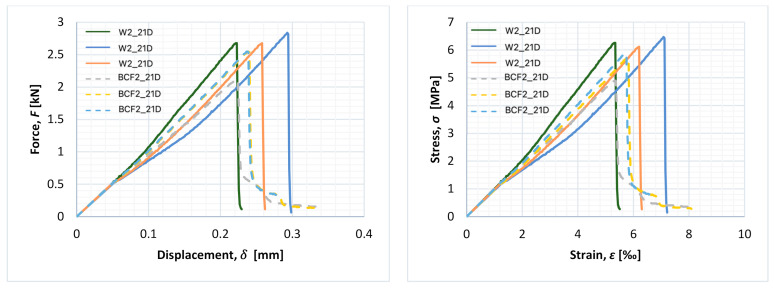
Force–displacement and stress–strain curves for the entire test duration for each specimen of series W2_21D (continuous line) and BCF2_21D (dashed line).

Figure 7, Figure 8 and Figure 9 present force–displacement and stress–strain curves obtained during the tests for each of the three specimens in the matching series (with and without fibres).

A rapid decrease in force after achieving the maximum value is typical for non-reinforced mortar, while the bridging effect in fibre-reinforced mortars might result in the smooth decrease in force with the continuous growth of displacement after cracking. In the case of non-reinforced mortar, after achieving the maximum value of force, cracks occurred and then immediately failure of the specimen (with rapid a decrease in force value). In the case of fibre-reinforced mortars, after achieving the maximum value of load, the cementitious matrix degraded, but the fibres formed a bridge between crack edges and were still able to transfer a reduced amount of the load. Even though the value of the load decreased as the test went on, it did not decrease rapidly. It should be underlined, however, that this effect was not noticed for every fibre-reinforced specimen in this research.

For series BCF1_21D and BCF2_1D, lower values of the maximum value of force during the flexural stage were obtained in comparison to reference mortars without fibres, while post-critical residual strength due to the bridging effect could be observed in the dashed curves, as shown in Figure 7 and Figure 8 (a slight decrease in force with continuous growth of displacement). This could be beneficial in various applications and means that the specimen is capable of bearing reduced amounts of load with the increase in deformations after achieving the maximum value of the load.

Such a phenomenon was observed only for BCF cementitious mortars with lower values of flexural strength. Even though series BCF2_1D (Figure 8) and BCF2_21D (Figure 9) were the same in terms of mixture proportions, the bridging effect was observed only for specimens examined after 1 day (dashed line in Figure 8).

In the Table 5, the results of fracture energy calculations, which increased due to basalt fibre addition for series BCF1_21D and BCF2_1D, are presented.

## 5. Discussion

**Figure 10:**
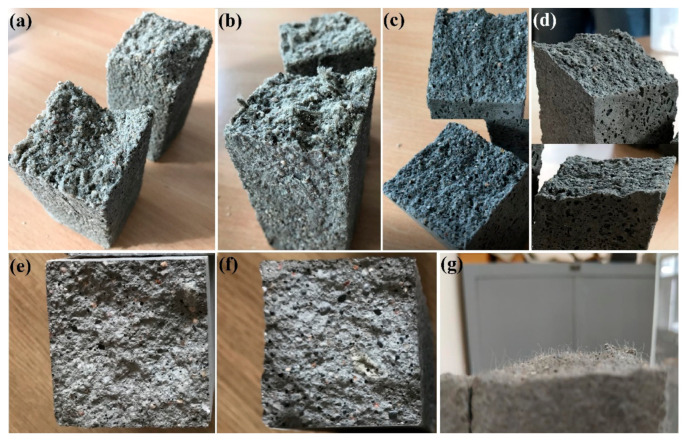
Damaged specimens of various series after flexural strength tests: (**a**) W1_21D, (**b**) BCF1_21D, (**c**) W2_21D, (**d**) BCF2_21D, (**e**) W3_28D, (**f**) BCF3_28D and (**g**) PP3_28D.

The addition of basalt fibres to cementitious mortar caused the fresh mixture to be much denser, while the hardened matrix had a more porous structure (see Figure 10). Such a change in the structure of the hardened matrix was also easily visible when analysing density results, because in all cases, the density of mortars with basalt fibre addition was lower compared to reference series without fibres (Figure 5 and Figure 6). Problems related to workability with fresh mortar with fibre addition include the increased porosity of hardened mortar, which should be taken into account when analysing possible applications of such material and have been underlined in previous studies by other authors [4,6,32,38,39,40].

The main aim of the presented research was to assess the influence of basalt fibres and other factors on flexural strength. The results of the series of mortars tested first revealed interesting differences in terms of their post-critical behaviour and, therefore, the closer analysis of this phenomena was performed as presented in this article. A bridging effect was noticed by means of routine flexural testing without any additional standard tests.

As there is no available international (ISO) standard concerning the bridging effect in fibre-reinforced cementitious mortars, further tests of such material could be designed as an analogy to regional standards for fibre-reinforced concrete [61,62]. Such experimental investigations, performed on notched beams, may also provide interesting data in reference to experimental results from presented research.

Furthermore, as there are no specific standard methods concerning the bridging effect of cementitious mortars that can be used during the introduction of such material to the market, experimental data provided in this article could be a useful contribution to further standardization works.

When analysing the results of flexural tests carried out on hardened specimens, the following main topics need to be considered:

(1) Influence of mixture proportions (water to cement ratio and addition of micro-silica and plasticiser);
(2) Issues related to specimen drying, the reduction of water inside the pores and other curing condition parameters (e.g., time);
(3) Comparison to reference polypropylene fibres.

Table 6 summarises all the results obtained in this research programme.

The addition of basalt fibres to cementitious mortars resulted in increasing flexural strength for one of the series (BCF3_28D), but the growth was only at a level of 8.5% in comparison to mortars without fibres. The increasing flexural strength value due to basalt fibre addition was obtained for the series with the lowest value of *w/c* ratio, which equals 0.5 (Figure 4). The trend was reversed for specimens with the same mixture proportions but dried out until they reached a stable mass. In that case, mortars with basalt fibres had a flexural strength lower than 0.4% and no bridging effect.

After tests of series with a *w/c* ratio equal to 0.5 and fibre content of 1% by binder mass, it was noticed that fibres were hardly visible on the cracked surface of the specimens (especially when compared to the polypropylene fibres that were easily visible on the cracked surface—Figure 10).

Mortars with a lower amount of cement and higher dosage of fibres were designed in order to analyse how the addition of fibres in a very high amount influences the appearance of the cracked surface after tests and how it changes various parameters obtained from flexural tests. A lower mass of cement, however, was supposed to reduce alkali influence, especially destructive for this type of fibre. Finally, on the basis of these preliminary investigations, authors plan to perform an experimental programme in the future to obtain a wider view of the influence of alkali.

Although a loss of flexural strength was observed in the series with mixture proportions designed in the manner described above, the addition of fibres appeared to change the post-critical behaviour of the specimens in some cases, especially for specimens with a short time of conditioning and low cement amount. The negative influence of basalt chopped fibres on the flexural strength of cementitious composites has been noticed in other studies as well [63]. However, in those cases, other parameters (such as flexural toughness and impact strength) increased. A similar observation was made in this study (fracture energy increased up to 143.3% along with a decrease in flexural strength; see Table 5), but only for series BCF1_21D and BCF2_1D. Mortars in both series had a relatively high *w/c* ratio and low amount of cement (200 kg/m^3^ and 300 kg/m^3^).

Moreover, when comparing the behaviour of these series, the bridging effect occurred only for specimens tested after 1 day from demoulding and did not occur after 21 days (even though the mixture proportions were the same), probably because the highly alkaline environment inside the cementitious matrix strongly influenced basalt fibre performance, especially after longer curing. Similar problems have been already reported in previously published studies of other authors, for instance [3,40,47].

Drying out specimens until stable mass caused a marked improvement in flexural strength, regardless of mortar type. The increase in flexural strength value was at a level of 86.0% in the case of mortars without fibres, at 70.7% for mortars with basalt fibres and at 74.9% for mortars with polypropylene fibres (see flexural strength values in Table 6). The increase in the flexural strength of specimens submitted to drying prior to testing might be related to the reduced amount of free water and water vapour inside the pores, as most of this water had evaporated or been used in hydration processes before testing. The influence of saturation state (dry conditions, partial saturation and full saturation) on the static mechanical properties of concrete was the subject of analysis in [64]. The approximate amount of water loss due to drying prior to flexural tests can be noticed in this research by comparing density values (see Table 6—density values are 3.8–5.2% lower for dried out specimens).

Additionally, hydration processes are known to be significantly accelerated by temperature elevation [65,66,67], which may be the cause of such flexural strength increase. The research provided in [65,66,67] has already proved that the exposure to a high temperature can increase the pace of hydration; however, the influence of such acceleration performed at an early stage on long-term strength can be contradictory. Porosity and SEM analyses are recommended in future research projects to better visualize changes in the structure of dried out specimens. It is also worth mentioning that mortars without fibres demonstrated a higher increase in flexural strength due to temperature influence than specimens with fibres (regardless of fibre type). However, drying out specimens did not influence the presence of the bridging effect.

Although the bridging effect was not noticed for series BCF3_28D and BCF3_28D_70, the increase in flexural strength in the case of non-dried specimens gives reason to suspect that the degradation of fibres was slower in that case. Further analyses of basalt fibre exposure to an alkali environment under various humidity and temperature conditions may give interesting results.

When comparing the addition of the same mass of basalt and polypropylene fibres (1% by binder mass–series BBCF3_28D, PP3_28D, BBCF3_28D_70 and PP3_28D_70), higher values of flexural strength were obtained for basalt-reinforced mortars (Table 6, Figure 6). The bridging effect, however, was obtained only for polypropylene and not for basalt fibres in that case.

Series with designations BCF1_21D and PP3_28D had a similar volumetric dosage of fibres (0.48% and 0.54%). Values of flexural strength cannot be directly compared, as these mortars differed significantly in terms of *w/c* ratio and duration of conditioning. However, for both of them, flexural tests did not end in rupture mode as a result of bridging effect occurrence. It is worth mentioning that in that case, the dosage of basalt fibres (6.2% by binder’s mass) was significantly higher than suggested by the literature (1–3%), which proves the necessity of further tests of this material.

The conducted research provided important experimental data on the flexural performance of relatively new material, namely, basalt fibre-reinforced cementitious mortar. The article highlights its main limitations (such as low alkali resistance or increased porosity) and advantages (bridging action occurrence or flexural strengthening depending on mixture proportions and curing conditions), so that the application potential of this material could be determined.

Available data in the up-to-date literature present a limited amount of experimental investigation results suggesting proper mixture proportions for cementitious mortars with this particular type of fibre (basalt). Additional information on this matter will be useful in planning further investigations with regard to defining suitable applicational fields. The authors of this article relied, however, on experience in basalt-reinforced concrete research programmes carried out to date and cementitious mortars with other types of fibres.

End users might benefit from results of this programme, as such mortar appears to be suitable for applications, where flexural strength itself is not so important, while flexural toughness plays a significant role (e.g., renovative mortars). However, problems related to alkali influence on basalt fibres have been underlined and should not be neglected when designing and using such mortar.

## 6. Summary and Conclusions

The results of flexural tests of basalt fibre-reinforced cementitious mortars in terms of flexural strength and the occurrence of the bridging effect are presented and compared to the reference specimens without fibres and with the addition of more common, polypropylene fibres. Various mixture proportions and curing conditions were analysed. The main variables in the mixture proportions were water to cement ratio, micro-silica and plasticiser addition and fibre dosage. Curing conditions were defined by temperature, humidity and time. The main conclusions are presented below.

(1) The bridging effect is among the biggest advantages of fibre-reinforced cementitious materials. In the case of cementitious mortars reinforced with basalt fibres examined in this research programme, however, this was observed only for mortars with a relatively low amount of cement and high *w/c* ratio. Additionally, for the series with the same mixture proportions, the effect occurred in tests carried out after 1 day from demoulding, while it did not occur after 21 days from demoulding, probably due to the fact that the highly alkaline environment inside the cementitious matrix strongly influences basalt fibre performance, especially after longer periods of time.
(2) Mortars with the addition of basalt fibres demonstrated higher values of flexural strength than reference mortars with polypropylene fibres (the same percentage dosage by the mass of the binder). Nevertheless, the bridging effect in that case was obtained only for polypropylene fibres.
(3) Drying out specimens until they reached a stable mass caused an improvement of flexural strength at a range of 70% to 86%; however, it did not influence the bridging effect.

The influence of the alkali environment on basalt fibre performance should be the main concern of future experimental investigations on cementitious mortars with basalt fibres, concentrating as much as possible on protection measures (e.g., protective coatings [40]). It is also recommended to investigate series of mixtures with the same volumetric percentage of fibre addition so the differences between densities of fibres can be omitted. Additionally, the workability of the fresh mixture and the porosity of hardened mortar should be taken into account. The authors presented their observations related to the workability of fresh mortar and the porosity of hardened specimens with the addition of basalt fibres, as well as the alkali resistance of fibres, which should be more carefully considered in future experimental investigations. Tests on notched beams, similar to tests performed on concrete specimens accordingly to standards [61,62], are also recommended.

## Figures and Tables

**Table 1:** Chemical composition of basalt fibres as declared by the manufacturer—percentage of overall mass [%].

	SiO_2_	Al_2_O_3_	Fe_2_O_3_	CaO	MgO	TiO_2_	Na_2_O	Others
Minima	45	12	5	6	3	0.9	2.5	2.0
Maxima	60	19	15	12	7	2.0	6.0	3.5

**Table 2:** Chemical composition of micro-silica as declared by the manufacturer.

	SiO_2_	Al_2_O_3_	CaO + MgO	K_2_O + Na_2_O	C
Percentage of overall mass (%)	89.00	0.49	2.30	3.62	2.5

**Table 3:** Composition of the experimental mortars.

Series Symbol ^1^	Cement (kg/m^3^)	Sand (kg/m^3^)	Water (kg/m^3^)	Micro-Silica (kg/m^3^)	Plasticiser (kg/m^3^)	BCF Fibres			PP Fibres			*w/c* Ratio ^2^
						By Weight (kg/m^3^)	Weight Percentage by Binder Mass (%)	Percentage of Overall Volume (%)	By Weight (kg/m^3^)	Weight Percentage by Binder Mass (%)	Percentage of Overall Volume (%)	
W1_21D	200	1862	234.5	–	2.8	–	–	–	–	–	–	1.17
BCF1_21D	200	1862	234.5	–	2.8	12.4	6.2	0.46	–	–	–	1.17
W2_1D W2_21D	300	1750	215	30	3.0	–	–	–	–	–	–	0.72
BCF2_1D BCF2_21D	300	1750	215	30	3.0	9.0	3.0	0.33	–	–	–	0.72
W3_28D W3_28D_70	500	1500	250	50	5.0	–	–	–	–	–	–	0.50
BCF3_28D BCF3_28D_70	500	1500	250	50	5.0	5.0	1.0	0.18	–	–	–	0.50
PP3_28D PP3_28D_70	500	1500	250	50	5.0	–	–	–	5.0	1.0	0.54	0.50

^1^ Code explanation: W—mortars without fibres; BCF—mortars with BCF fibres; PP—mortars with PP fibres; 1, 2, 3—mixture type in terms of *w/c* ratio and presence of additions; 1D, 21D, 28D—storage time before testing (1 day, 21 days, 28 days); 70—specimens dried out to constant weight in a temperature of 70 °C; ^2^ *w/c* ratio–water to cement ratio, where: *w*—mass of water with plasticiser; *c*—mass of cement.

**Table 4:** Conditions and storage time for each series of samples.

Conditioning Description	Series Symbol											
	W1_21D	BCF1_21D	W2_1D	BCF2_1D	W2_21D	BCF2_21D	W3_28D	BCF3_28D	PP3_28D	W3_28D_70	BCF3_28D_70	PP3_28D_70
– specimens stored in forms—air temperature in the room: 20 °C	0th–1st	0th–1st	0th–1st	0th–1st	0th–1st	0th–1st	0th–2nd	0th–2nd	0th–2nd	0th–2nd	0th–2nd	0th–2nd
– specimens stored in air saturated with water vapour at a level not less than 95%—air temperature: 20 °C	2nd–21st	2nd–21st	–	–	2nd–21st	2n–21st	–	–	–	–	–	–
– specimens stored in water—water temperature: 20 °C	–	–	–	–	–	–	3rd–21st	3rd–21st	3rd–21st	3rd–21st	3rd–21st	3rd–21st
– air-dry conditions—air temperature in the room: 20 °C	–	–	–	–	–	–	22nd–28th	22nd–28th	22nd–28th	–	–	–
– specimens stored inside the dryer—air temperature inside the dryer: 70 °C	–	–	–	–	–	–	–	–	–	22nd–28th	22nd–28th	22nd–28th

**Table 5:** Mean value of fracture energy, *G_F_* (N/m).

	W1_21D/BCF1_21D	W2_1D/BCF2_1D	W2_21D/BCF2_21D
without fibres	56.93	41.91	206.19
with fibres	85.51	101.95	174.86
increase due to fibres addition	+50.2%	+143.3%	−15.2%

**Table 6:** Summary of all the results from this research programme: flexural strength and density of mortars and occurrence of bridging effect.

Series Symbol	Flexural Strength		Density		Fracture Energy
	Mean Value (MPa)	Standard Deviation (MPa)	Mean Value (kg/m^3^)	Standard Deviation (kg/m^3^)	Mean Value (N/m)
W1_21D	2.70	0.006	2216.0	21.47	56.93
BCF1_21D ^1,2^	1.83	0.112	2051.4	21.38	85.51
W2_1D	2.28	0.064	2137.1	6.88	41.91
BCF2_1D ^1,2^	1.75	0.079	1962.4	14.11	101.95
W2_21D	6.27	0.083	2135.2	5.75	206.19
BCF2_21D	5.28	0.341	1949.6	4.23	174.86
W3_28D	7.43	0.260	2432.7	26.70	-
BCF3_28D	8.06	0.478	2329.5	17.76	-
PP3_28D ^1^	7.38	0.251	2224.7	22.34	-
W3_28D_70	13.82	0.855	2306.3	30.80	-
BCF3_28D_70	13.76	0.811	2240.0	23.03	-
PP3_28D_70 ^1^	12.91	0.441	2111.0	13.91	-

^1^ No rupture end of the test due to bridging effect. ^2^ Confirmed increase in fracture energy (see Table 5).

## Data Availability

Data supporting findings of this study is contained within this article.

## References

[1] Banthia N., Zanotti C., Sappakittipakorn M. (2014). Sustainable fiber reinforced concrete for repair applications. Constr. Build. Mater..

[2] Kulesza M., Dębski D., Fangrat J., Michalak J. (2020). Effect of redispersible polymer powders on selected mechanical properties of thin-bed cementitious mortars. Cem. Wapno Beton.

[3] Puertas F., Amat T., Fernández-Jiménez A., Vázquez T. (2003). Mechanical and durable behaviour of alkaline cement mortars reinforced with polypropylene fibres. Cem. Concr. Res..

[4] Jiang C., Huang S., Zhu Y., Lin Y., Chen D. (2016). Effect of polypropylene and basalt fiber on the behavior of mortars for repair applications. Adv. Mater. Sci. Eng..

[5] Iorfida A., Verre S., Candamano S., Ombres L. (2018). Tensile and direct shear responses of basalt-fibre reinforced mortar based materials. RILEM Bookseries.

[6] Asprone D., Cadoni E., Iucolano F., Prota A. (2014). Analysis of the strain-rate behavior of a basalt fiber reinforced natural hydraulic mortar. Cem. Concr. Compos..

[7] Santarelli M.L., Sbardella F., Zuena M., Tirillò J., Sarasini F. (2014). Basalt fiber reinforced natural hydraulic lime mortars: A potential bio-based material for restoration. Mater. Des..

[8] Rawat S., Narula R., Upasani N., Muthukumar G. (2020). A relook on dosage of basalt chopped fibres and its influence on characteristics of concrete. Advances in Sustainable Construction Materials and Geotechnical Engineering.

[9] Li V.C., Lin Z.L., Matsumoto T. (1998). Influence of fiber bridging on structural size-effect. Int. J. Solids Struct..

[10] Abed F., Alhafiz A.R. (2019). Effect of basalt fibers on the flexural behavior of concrete beams reinforced with BFRP bars. Compos. Struct..

[11] Tiberti G., Minelli F., Plizzari G. (2015). Cracking behavior in reinforced concrete members with steel fibers: A comprehensive experimental study. Cem. Concr. Res..

[12] Deng M., Han J., Liu H., Qin M., Liang X. (2015). Analysis of Compressive Toughness and Deformability of High Ductile Fiber Reinforced Concrete. Adv. Mater. Sci. Eng..

[13] Chang J., Cui K., Zhang Y. (2020). Effect of hybrid steel fibers on the mechanical performances and microstructure of sulphoaluminate cement-based reactive powder concrete. Constr. Build. Mater..

[14] Amran M., Fediuk R., Vatin N., Lee Y.H., Murali G., Ozbakkaloglu T., Klyuev S., Alabduljabber H. (2020). Fibre-reinforced foamed concretes: A review. Materials.

[15] Paul S.C., van Zijl G.P.A.G., Šavija B. (2020). Effect of fibers on durability of concrete: A practical review. Materials.

[16] Li V.C., Stang H., Krenchel H. (1993). Micromechanics of crack bridging in fibre-reinforced concrete. Mater. Struct..

[17] Chiaia B., Fantilli A.P., Vallini P. (2009). Evaluation of crack width in FRC structures and application to tunnel linings. Mater. Struct. Constr..

[18] Zheng D., Song W., Fu J., Xue G., Li J., Cao S. (2020). Research on mechanical characteristics, fractal dimension and internal structure of fiber reinforced concrete under uniaxial compression. Constr. Build. Mater..

[19] Arshad S., Sharif M.B., Irfan-ul-Hassan M., Khan M., Zhang J.L. (2020). Efficiency of Supplementary Cementitious Materials and Natural Fiber on Mechanical Performance of Concrete. Arab. J. Sci. Eng..

[20] Chen M., Zhong H., Wang H., Zhang M. (2020). Behaviour of recycled tyre polymer fibre reinforced concrete under dynamic splitting tension. Cem. Concr. Compos..

[21] Lau C.K., Chegenizadeh A., Htut T.N.S., Nikraz H. (2020). Performance of the steel fibre reinforced rigid concrete pavement in fatigue. Buildings.

[22] Zareei S.A., Ameri F., Bahrami N., Shoaei P., Musaeei H.R., Nurian F. (2019). Green high strength concrete containing recycled waste ceramic aggregates and waste carpet fibers: Mechanical, durability, and microstructural properties. J. Build. Eng..

[23] Carvalho M.R., Barros J.A.O., Zhang Y., Dias-da-Costa D. (2020). A computational model for simulation of steel fibre reinforced concrete with explicit fibres and cracks. Comput. Methods Appl. Mech. Eng..

[24] Tailhan J.L., Rossi P., Daviau-Desnoyers D. (2015). Probabilistic numerical modelling of cracking in steel fibre reinforced concretes (SFRC) structures. Cem. Concr. Compos..

[25] Tarasovs S., Krūmiņš J., Tamužs V. (2016). Modelling of the fracture toughness anisotropy in fiber reinforced concrete. Frattura ed Integrità Strutturale.

[26] Chi Y., Xu L., Yu H.S. (2014). Constitutive modeling of steel-polypropylene hybrid fiber reinforced concrete using a non-associated plasticity and its numerical implementation. Compos. Struct..

[27] Bernardi P., Cerioni R., Michelini E. (2013). Analysis of post-cracking stage in SFRC elements through a non-linear numerical approach. Eng. Fract. Mech..

[28] Ito H., Watanabe K., Todoroki S., Suemori H., Shinjyo R. (2018). Study on performance of PVA fiber reinforced concrete exposed for 10 years to seawater spray. J. Adv. Concr. Technol..

[29] Kim M.O., Bordelon A.C., Lee N.K. (2017). Early-age crack widths of thin fiber reinforced concrete overlays subjected to temperature gradients. Constr. Build. Mater..

[30] Mohammed A.A., Manalo A.C., Ferdous W., Zhuge Y., Vijay P.V., Pettigrew J. (2020). Experimental and numerical evaluations on the behaviour of structures repaired using prefabricated FRP composites jacket. Eng. Struct..

[31] Al-Rubaye M., Manalo A., Alajarmeh O., Ferdous W., Lokuge W., Benmokrane B., Edoo A. (2020). Flexural behaviour of concrete slabs reinforced with GFRP bars and hollow composite reinforcing systems. Compos. Struct..

[32] Jiang C., Fan K., Wu F., Chen D. (2014). Experimental study on the mechanical properties and microstructure of chopped basalt fibre reinforced concrete. Mater. Des..

[33] Dias D.P., Thaumaturgo C. (2005). Fracture toughness of geopolymeric concretes reinforced with basalt fibers. Cem. Concr. Compos..

[34] Borinaga-Treviño R., Orbe A., Norambuena-Contreras J., Canales J. (2018). Effect of microwave heating damage on the electrical, thermal and mechanical properties of fibre-reinforced cement mortars. Constr. Build. Mater..

[35] Bilotta A., Lignola G.P. (2020). Effects of Defects on Bond Behavior of Fiber Reinforced Cementitious Matrix Materials. Materials.

[36] Padalu P.K.V.R., Singh Y., Das S. (2018). Efficacy of basalt fibre reinforced cement mortar composite for out-of-plane strengthening of unreinforced masonry. Constr. Build. Mater..

[37] Lignola G.P., Caggegi C., Ceroni F., de Santis S., Krajewski P., Lourenço P.B., Morganti M., Papanicolaou C., Pellegrino C., Prota A. (2017). Performance assessment of basalt FRCM for retrofit applications on masonry. Compos. Part B Eng..

[38] Li L.G., Zeng K.L., Ouyang Y., Kwan A.K.H. (2019). Basalt fibre-reinforced mortar: Rheology modelling based on water film thickness and fibre content. Constr. Build. Mater..

[39] Kabay N. (2014). Abrasion resistance and fracture energy of concretes with basalt fiber. Constr. Build. Mater..

[40] Lipatov Y.V., Gutnikov S.I., Manylov M.S., Zhukovskaya E.S., Lazoryak B.I. (2015). High alkali-resistant basalt fiber for reinforcing concrete. Mater. Des..

[41] Simões T., Costa H., Dias-da-Costa D., Júlio E. (2018). Influence of type and dosage of micro-fibres on the physical properties of fibre reinforced mortar matrixes. Constr. Build. Mater..

[42] Li L.G., Zhuo H.X., Zhu J., Kwan A.K.H. (2019). Packing density of mortar containing polypropylene, carbon or basalt fibres under dry and wet conditions. Powder Technol..

[43] Barnat-Hunek D., Łagód G., Fic S., Jarosz-Hadam M. (2018). Effect of polysiloxanes on roughness and durability of basalt fibres-reinforced cement mortar. Polymers.

[44] Atiyeh M., Aydin E. (2020). Carbon-Fiber Enriched Cement-Based Composites for Better Sustainability. Materials.

[45] Pereira M.V., Fujiyama R., Darwish F., Alves G.T. (2015). On the strengthening of cement mortar by natural fibers. Mater. Res..

[46] Pareek K., Saha P. Basalt fiber and its composites: An overview. Proceedings of the National Conference on Advances in Structural Technologies (CoAST-2019).

[47] Li Z., Ma J., Ma H., Xu X. (2018). Properties and applications of basalt fiber and its composites. IOP Conf. Ser. Earth Environ. Sci..

[48] Cheng Y., Li L., Zhou P., Zhand Y., Liu H. (2019). Multi-Objective Optimization Design and Test of Compound Diatomite and Basalt Fibre Asphalt Mixture. Materials.

[49] Zhou H., Jia B., Huang H., Mou Y. (2020). Experimental study on basic mechanical properties of basalt fiber reinforced concrete. Materials.

[50] Hanafi M., Aydin E., Ekinci A. (2020). Engineering properties of basalt fiber-reinforced bottom ash cement paste composites. Materials.

[51] Ralegaonkar R., Gavali H., Aswath P., Abolmaali S. (2018). Application of chopped basalt fibers in reinforced mortar: A review. Constr. Build. Mater..

[52] van Gemert D., Czarnecki L., Maultzsch M., Schorn H., Beeldens A., Łukowski P., Knapen E. (2005). Cement concrete and concrete-polymer composites: Two merging worlds: A report from 11th ICPIC Congress in Berlin, 2004. Cem. Concr. Compos..

[53] Kubissa W., Simon T., Jaskulski R., Reiterman P., Supera M. (2017). Ecological high performance concrete. Procedia Eng..

[54] Ipbüker C., Nulk H., Gulik V., Biland A., Tkaczyk A.H. (2015). Radiation shielding properties of a novel cement-basalt mixture for nuclear energy applications. Nucl. Eng. Des..

[55] Lee J.J., Song J., Kim H. (2014). Chemical stability of basalt fiber in alkaline solution. Fibers Polym..

[56] Grzybowski M., Wang J., Karihaloo B. (1996). Technical Report: Single and Multiple Bridged Cracks: Application to Fibre-Reinforced Solids.

[57] Yonggui W., Shuaipeng L., Hughes P., Yuhui F. (2020). Mechanical properties and microstructure of basalt fibre and nano-silica reinforced recycled concrete after exposure to elevated temperatures. Constr. Build. Mater..

[58] Kooshafar M. (2017). Influential Mechanisms and Potential Applications of Nano-Silicas in Cement Composites. Civ. Eng. Infrastruct. J..

[59] European Committee for Standardization (2016). CEN-EN 196-1:2016 Methods of Testing Cement. Determination of Strength.

[60] Kozłowski M., Kadela M., Kukiełka A. (2015). Fracture energy of foamed concrete based on three-point bending test on notched beams. Procedia Eng..

[61] America Concrete Institute (2009). ACI 446 Fracture Toughness Testing of Concrete.

[62] British Standards Institution (2005). EN 14651 Test Method for Metallic Fibred Concrete—Measuring the Flexural Tensile Strength (Limit of Proportionality (LOP), Residual).

[63] Ramakrishnan V., Tolmare N., Brik V. (1998). Performance Evaluation of 3-D Basalt Fiber Reinforced Concrete and Basalt Rod Reinforced Concrete. Innovations Deserving Exploratory Analysis Programs (IDEA) Program Final Report.

[64] Zhang G., Li X., Li Z. (2019). Experimental Study on Static Mechanical Properties and Moisture Contents of Concrete Under Water Environment. Sustainability.

[65] Deschner F., Lothenbach B., Winnefeld F., Neubauer J. (2013). Effect of temperature on the hydration of Portland cement blended with siliceous fly ash. Cem. Concr. Res..

[66] Martínez-Ramírez S., Frías M. (2009). The effect of curing temperature on white cement hydration. Constr. Build. Mater..

[67] del Bosque I.F.S., Martínez-Ramírez S., Blanco-Varela M. (2013). Combined effect of amorphous nanosilica and temperature on white Portland cement hydration. Ind. Eng. Chem. Res..

